# Development and application of a UHPLC-MS/MS method for the simultaneous determination of firmonertinib and its main metabolite AST-5902 in rat plasma: a study on the *in vivo* drug interaction between firmonertinib and paxlovid

**DOI:** 10.3389/fphar.2025.1570206

**Published:** 2025-05-12

**Authors:** Peng-Fei Tang, Su-Su Bao, Wei-Fei Xie, Zhong-Xiang Xiao, Xue-Meng Wu, Hong-Lei Ge

**Affiliations:** ^1^ Department of Clinical Pharmacy Room, Affiliated Yueqing Hospital of Wenzhou Medical University, Wenzhou, Zhejiang, China; ^2^ Department of Hematology and Chemotherapy, Affiliated Yueqing Hospital of Wenzhou Medical University, Wenzhou, Zhejiang, China; ^3^ Department of General Department, Market Supervision Administration of Yueqing City, Wenzhou, Zhejiang, China

**Keywords:** firmonertinib, drug interactions, UHPLC-MS/MS, pharmacokinetics, paxlovid

## Abstract

Due to the potential occurrence of drug interactions, the combined application of firmonertinib and paxlovid carries a relatively high risk. Nevertheless, as of now, there has been no comprehensive research on the interaction between firmonertinib and paxlovid. Our aim was to establish and validate an accurate, stable, rapid and simple UPLC-MS/MS method for the simultaneous determination of firmonertinib and its metabolite AST-5902 in rat plasma, which was applied to the study of the *in vivo* interaction between firmonertinib and paxlovid. Gefitinib was selected as the internal standard. After protein precipitation of the plasma samples with acetonitrile, the separation was carried out on a Shimadzu LC-20AT UHPLC. The chromatographic column was a Shim-pack Volex PFPP column (50 mm × 2.1 mm, 1.8 μm), and the mobile phase was composed of 0.1% formic acid - water and 0.1% formic acid - methanol. Mass spectrometry detection was performed using a Shimadzu 8,040 mass spectrometer in ESI+ and MRM mode. The precision, accuracy, recovery and matrix effect of this method were detected. The linearity of the method and the stability of the samples were assessed. Subsequently, the method was applied to the study of the interaction between firmonertinib and paxlovid. The parent ions and typical fragment ions of firmonertinib, AST-5902 and IS are respectively m/z 569.25 → 72.15, m/z 555.50 → 498.10 and m/z 447.25→ 128.20. The selectivity, specificity, linearity, recovery, matrix effect, accuracy and precision of the method and the stability of the samples were all adequately verified. The results of drug interaction showed that when firmonertinib was combined with paxlovid, the AUC and Cmax of firmonertinib were significantly increased, while the AUC, Tmax, and Cmax of AST-5902 were significantly decreased. The established UHPLC-MS/MS detection method is accurate, stable, rapid and simple. Paxlovid exhibit a significant inhibitory effect on the metabolism of firmonertinib in rats.

## 1 Introduction

Coronavirus disease 2019 (COVID-19), triggered by the severe acute respiratory syndrome coronavirus 2 (SARS-CoV-2), has been rapidly spreading globally since 2019, posing a great threat to public physical and mental health (Umakanthan et al., 2020). According to the data from the World Health Organization (WHO), as of the present time, over 777 million individuals globally have been infected with COVID-19, giving rise to more than 7 million fatalities (Sarrazin and Cáceres, 2025). In the face of such a vast number of infected individuals and the constant emergence of new variants strains, seeking practical and effective treatment approaches has become the top priority (Tao et al., 2021; Shrestha et al., 2022). A variety of treatment methods have been previously reported, including various existing antiviral drugs, immunomodulators, monoclonal antibodies, etc., (Drożdżal et al., 2021; Parums, 2022). However, these treatment approaches exhibit certain limitations to varying extents, including uncertain therapeutic efficacy, a high incidence of adverse reactions, the requirement for inpatient administration, and substantial costs (Yuan et al., 2023). These shortcomings have impeded their widespread clinical application. Therefore, there is an urgent need for an oral drug with definite therapeutic effects and mild adverse reactions to treat COVID-19.

The novel oral antiviral drug “Paxlovid” exhibits excellent therapeutic efficacy against COVID-19 (Amani and Amani, 2023). Paxlovid is composed of nirmatrelvir and ritonavir. Nirmatrelvir is an inhibitor specifically targeting the 3CLpro protease of the SARS-CoV-2 virus (Yang et al., 2022). Ritonavir is an aspartic protease inhibitor of human immunodeficiency virus-1 (HIV-1) and a potent CYP3A4 inhibitor (Lea and Faulds, 1996). When combined with nirmatrelvir, it can inhibit the metabolism of nirmatrelvir, thereby increasing the blood concentration and retention time of nirmatrelvir in the body and enhancing the therapeutic effect of nirmatrelvir (Lamb, 2022). Paxlovid has been approved by the US FDA for the treatment of adult and adolescent patients with mild to moderate COVID-19 who have risk factors for progression to severe disease (Reina and Iglesias, 2022). Owing to the application of ritonavir, when Paxlovid is combined with other drugs metabolized by CYP3A4 (such as the majority of anti-tumor targeted drugs, etc.), drug-drug interactions (DDI) might take place (Marzolini et al., 2022; Tang et al., 2022; Li C. et al., 2023).

The global COVID-19 pandemic has posed significant challenges to the healthcare system, especially for patients with underlying diseases (Zhang et al., 2022; Oláh, 2023). Among these, non-small cell lung cancer (NSCLC) patients represent a particularly vulnerable group (Huang et al., 2024). Patients diagnosed with NSCLC have a higher risk of being infected with COVID-19 than normal individuals, particularly among elderly patients (Sebastian et al., 2024). Furthermore, lung cancer patients are generally at higher risk of severe outcomes when infected with COVID-19, likely due to factors such as immune suppression from cancer treatments, pre-existing lung damage, and comorbidities (Addeo and Friedlaender, 2020; Kahya et al., 2021; Provencio et al., 2021; Elkrief et al., 2022). Therefore, for patients with both NSCLC and COVID-19, the situation becomes even more complex (Piper-Vallillo et al., 2021). Early-stage NSCLC typically manifests as non-specific symptoms such as mild coughing and fatigue, which are prone to be misidentified as common respiratory disorders (such as colds and bronchitis), and the presence of COVID-19 symptoms may further obscure the diagnosis of NSCLC (Metelmann et al., 2023; Orelaru et al., 2023). This dual diagnosis creates a pressing need to understand better how drugs used for the treatment of these two conditions interact with each other, potentially affecting drug efficacy and safety (Anwar et al., 2023).

**FIGURE 1:**
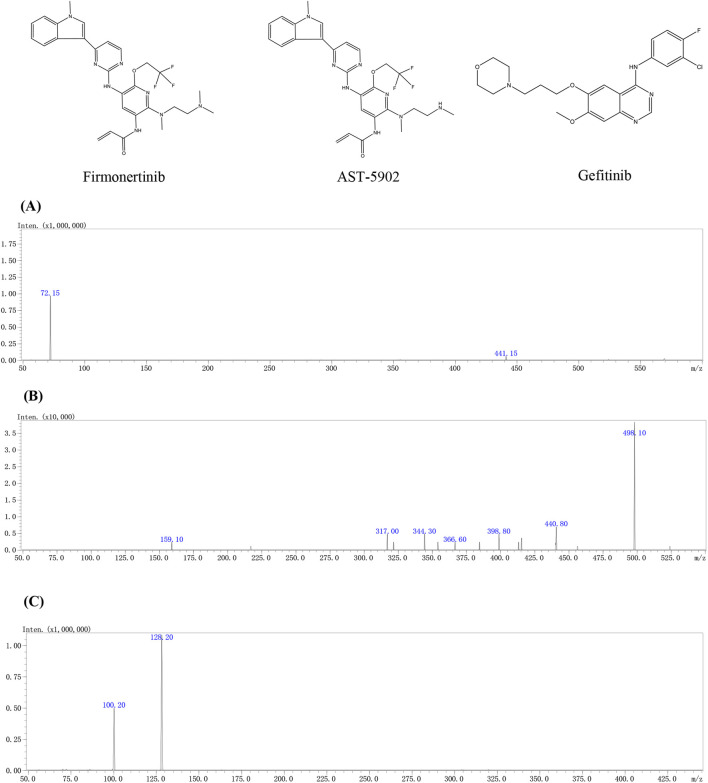
The chemical structures and mass spectra of firmonertinib **(A)**, AST-5902 **(B)** and gefitinib (IS) **(C)** in the present study.

Firmonertinib, a third-generation EGFR tyrosine kinase inhibitor (TKI), is widely used in treating NSCLC patients with EGFR mutations (Shi et al., 2022; Chen et al., 2023; Ding et al., 2024). The results of *in vitro* experiments showed that firmonertinib is metabolized mainly by CYP3A4 to the active N-demethylated metabolite AST-5902 (Figure 1) (Liu et al., 2020). Therefore, it is necessary to consider the potential DDIs that may occur when firmonertinib is used in combination with CYP3A4 inhibitors or inducers. Previous clinical studies have indicated that when firmonertinib is combined with itraconazole (a potent CYP3A4 inhibitor), the metabolism of firmonertinib is severely inhibited, and the exposure significantly increases (Heng et al., 2021). On the other hand, Paxlovid, a combination of nirmatrelvir and ritonavir, is used to treat COVID-19. Ritonavir is a potent CYP3A4 inhibitor, which can change the metabolism of drugs that are substrates of this enzyme, such as firmonertinib (Bege and Borbás, 2024). When firmonertinib is used in combination with Paxlovid, close attention should be paid to the changes in the pharmacokinetics of firmonertinib and the resulting variations in clinical efficacy or adverse reactions. However, there is a lack of comprehensive studies on the potential drug-drug interactions between these two therapies, especially in patients with co-existing NSCLC and COVID-19. Therefore, it is essential to assess the drug-drug interactions between Paxlovid and firmonertinib to support rational dose adjustments.

To investigate this critical issue, we first developed a highly sensitive and reliable UHPLC-MS/MS method for the simultaneous determination of firmonertinib and its main metabolite, AST-5902, in rat plasma. The goal of this study was to explore the *in vivo* drug interaction between Firmonertinib and Paxlovid, which could provide insights into the potential pharmacokinetic alterations when these two drugs are co-administered, especially in patients facing the dual burden of NSCLC and COVID-19. This study aims to provide more precise medication advice for the combined use of firmonertinib and Paxlovid, maximizing the prevention of treatment failure or adverse drug reactions.

## 2 Materials and methods

### 2.1 Chemicals and reagents

The chemicals and reagents used in the study were as follows: Firmonertinib mesylate (purity 99.0%), AST-5902 (purity 99.0%) and internal standard (IS) gefitinib (purity 99.0%) were obtained from Biorbyt Ltd. (Durham, North Carolina, United States). Nirmatrelvir (99.0% purity) and ritonavir (99.0% purity) were obtained from Chemleader Corporation (Shanghai, China). Mass spectrometry grade methanol, acetonitrile and 99% formic acid were provided by J&K Scientific Ltd. (Beijing, China). Ultrapure water (resistivity, 18.2 MΩ*cm, 25°C) was prepared using the Milli-Q synergy (UV) system (Millipore, MA, United States).

### 2.2 UHPLC–MS/MS detection method

The samples to be tested were separated on a Shimadzu Prominence LC-20A UHPLC system (Shimadzu Corp., Kyoto, Japan). Instrument control, data acquisition, and data analysis were all conducted using LabSolutions version 5.81 software (Shimadzu Corp., Kyoto, Japan). The separation was performed on a Shim-pack Volex PFPP column (50 mm × 2.1 mm, 1.8 μm) at 40°C. The sample volume was 3 µL per injection, the elution mobile phase consisted of 0.1% formic acid-water (A) and 0.1% formic acid-methanol (B) at a flow rate of 0.4 mL/min in a gradient elution mode with the following elution programme: Initially 0–0.5 min to maintain 10% B, 0.5–1 min to increase B to 80%, 1–2 min to maintain 80% B, 2–2.5 min to decrease B to 10%, and 2.5 min-3 min to maintain 10% B.

Samples were detected using a Shimadzu LC-MS 8040 (Shimadzu Corp., Tokyo, Japan). The instrument consists of an electrospray ionization (ESI) source, triple quadrupole mass analyser and a LabSolutions data acquisition station. The parameters of the ESI source are set as follows: capillary voltage: 4.5 kV, heating temperature 400°C, collision induced dissociation gas pressure: 230 kPa, atomising and drying gas flow rates: 3 L/min and 5 L/min respectively. In the positive ion mode, the product ion fragment with the highest signal intensity in the retention time range was used as the quantitative fragment and the fragment with the second strongest signal as the qualitative fragment to detect the target compound and internal standard in multiple reaction monitoring (MRM) mode. Table 1 provides detailed information on the MS parameters for firmonertinib, AST-5902 and IS.

**TABLE 1:** The parent ions and typical fragment ions of firmonertinib, AST-5902 and IS.

Analytes	Precursor ion (m/z)	Product ion 1 (m/z)	Collision energy 1 (V)	Product ion 2 (m/z)	Collision energy 2 (V)
Firmonertinib	569.25	72.15	29	441.15	27
AST-5902	555.50	498.10	25	440.80	27
IS	447.25	128.20	40	100.20	40

### 2.3 Calibration and QC samples preparation

One hundred milligram of firmonertinib, AST-5902 and IS were respectively dissolved in 10 mL of methanol to prepare the corresponding 10 mg/mL stock solutions of firmonertinib, AST-5902 and IS. The prepared stock solution was refrigerated at −40°C and bring to room temperature before use. The working solutions of firmonertinib and AST-5902 were prepared by diluting the stock solutions with methanol to the appropriate concentrations. Sample preparation for each point of the calibration curve was performed by adding 10 µL of firmonertinib and 10 µL of AST-5902 working solution to 80 µL of blank rat plasma. The final concentrations of the standard curves for firmonertinib and AST-5902 were respectively 0.1/0.05, 0.5/0.25, 1/0.5, 2.5/1.25, 5/2.5, 10/5, 50/25, 100/50, and 500/250 ng/mL. The stock solution of IS was diluted by methanol to prepare an IS working solution at a concentration of 300 ng/mL. Quality control (QC) samples were prepared by adding 10 µL of the appropriate concentration of firmonertinib or AST-5902 working solution to 90 µL of blank rat plasma. The QC samples of four concentrations (LLOQ, low, medium and high concentration) were prepared. The concentrations of the firmonertinib quality control samples are 0.1, 0.3, 40, and 400 ng/mL respectively, while the concentrations of AST-5902 are 0.05, 0.15, 20, and 200 ng/mL respectively. The prepared working solution, stock solution and QC samples were stored at −40°C and brought to room temperature before the experiment.

### 2.4 Sample preparation

Plasma specimens were stored in −80°C medical freezer and transferred to room temperature to thaw prior to analysis. After the plasma was completely thawed, 200 µl of acetonitrile and 20 µl of internal standard were added to 100 µl of plasma to precipitate the protein. The mixture was vortexed for 2 min and then centrifuged at 13,000 g for 10 min. The 100 µl of supernatant was transferred to a new centrifuge tube and diluted by adding 100 µl of ultrapure water, after slight mixing for 30 s, the mixture was analyzed using UHPLC-MS/MS.

### 2.5 Method validation

Prior to subsequent experiments using this method, the parameters of specificity, linearity, accuracy, precision, recovery matrix effect and stability of the method were validated according to the latest bioanalytical method validation guidance issued by the US FDA (FDA, 2018).

#### 2.5.1 Selectivity and specificity

Selectivity refers to the ability of an analytical method to distinguish and quantify target compounds in a mixture. Blank rat plasma, rat plasma spiked with firmonertinib and internal standard, and experimental rat plasma samples were determined using this method. The selectivity of the method was assessed by comparing the chromatograms of blank plasma, rat plasma spiked with the firmonertinib and internal standard and experimental rat plasma samples.

#### 2.5.2 Linearity and LLOQ

Standard curves for firmonertinib and AST-5902 were established by measuring nine different concentrations of firmonertinib/AST-5902 calibration samples on different 3 days. Standard curves were plotted by peak area ratios between analytes and internal standard (y-axis) against corresponding standard concentration (x-axis) and calculated using weighted (1/x^2^) least-square linear regression. Lower limit of quantification (LLOQ) is defined as the lowest concentration that can be reproducibly, precisely and accurately quantified. LLOQ requires a signal-to-noise ratio of at least 5:1, a precision of less than 20%, and an accuracy of 80%–120%.

#### 2.5.3 Recovery and matrix effect

The recovery and matrix effect of the present method were evaluated using high, medium and low three different concentrations of firmonertinib and AST-5902 quality control standards and blank rat plasma from six different rats. The ratios of peak area of blank plasma spiked with quality control standards and peak area of acetonitrile-treated plasma spiked with the same concentration of quality control standards was used to assess the recovery of present method. Matrix effect was derived by comparing peak areas of acetonitrile-treated plasma spiked with the quality control standards with peak areas of same concentration of quality control standards.

#### 2.5.4 Accuracy and precision

Low, medium, high and LLOQ four different concentrations rat plasma samples were determined using the present method on 1 day or three different days to assess the intra- and inter-day precision of the method. The precision of the method is considered good when both the relative error (RE%) and coefficient of variation (CV%) of the test results were below 15%, and the LLOQ was below 20%. The accuracy of the method was evaluated using the recoveries of rat plasma samples at low, medium, high and LLOQ four different concentrations. The accuracy of the method was considered good when the recoveries were in the range of 85%–115% and the LLOQ was in the range of 80%–120%.

#### 2.5.5 Stability

The stability of the method was assessed through the determination of low, medium and high concentration rat plasma quality control samples under various storage conditions. Six parallel samples per concentration were determined under experimental conditions (4 h at room temperature), short-term storage conditions (24 h at 4°C), long-term storage conditions (1 month at −40°C) and repeated freeze-thawing (three times). The stability of the samples was determined by the precision of measurement results, and the samples were considered stable when the CV% and RE% were both below 15%.

### 2.6 Drug interaction study

The experimental animals for pharmacokinetics were eighteen male Sprague-Dawley (SD) rats (6–8 weeks old), weighing 180–220 g, purchased from Beijing Vital River Laboratory Animal Technology Co., Ltd. All animal-related experimental operations and care were performed in accordance with the Guide for the Care and Use of Laboratory Animals and were approved by the Animal Research Ethics Committee of Wenzhou Medical University (wydw2022-0184). All SD rats were kept in an SPF animal laboratory and were provided with adequate feed and water until all rats reached the required body weight for the experiment. Firmonertinib, nirmatrelvir and ritonavir were dissolved in the pre-prepared 0.5% carboxymethyl cellulose sodium (CMC-Na). Eighteen SD rats were randomly and equally divided into three groups of six rats each. Group C rats (long-term administration group) were given 55 mg/kg nirmatrelvir and 20 mg/kg ritonavir by gavage every 12 h for 5 days. Groups B (single dose administration group) and A (control group) were also given the same amount of 0.5% CMC-Na every 12 h for 5 days. Prior to the pharmacokinetic experiments, all rats were fasted for 12 h, but drinking water was not prohibited. On the day of the experiment, 55 mg/kg nirmatrelvir and 20 mg/kg ritonavir were given once by gavage to rats in group B and group C. The same dose of 0.5% CMC-Na was given to group A rats. Half an hour later, all rats were given 7.2 mg/kg firmonertinib once by gavage, followed by 0.3 mL of blood taken from the tail vein at 1 h, 2 h, 4 h, 6 h, 8 h, 10 h, 12 h, 24 h, 48 h, 72 h. Blood was collected using heparin lithium-anticoagulant tubes and then centrifuged for 10 min at 13,000 rpm at 4°C. Subsequently, the upper plasma fraction was transferred to a clean centrifuge tube and placed in a −80°C refrigerator for storage until detection.

The pharmacokinetic parameters of firmonertinib and its metabolite AST-5902 were calculated using DAS 3.0 software according to non-compartment model. The pharmacokinetic parameters of three groups were analyzed by single factor analysis of variance using SPSS 28.0 software. A *p* value of less than 0.05 were considered significant difference between the two groups.

## 3 Results and discussion

### 3.1 Method development and optimization

#### 3.1.1 Chromatographic condition

**FIGURE 2:**
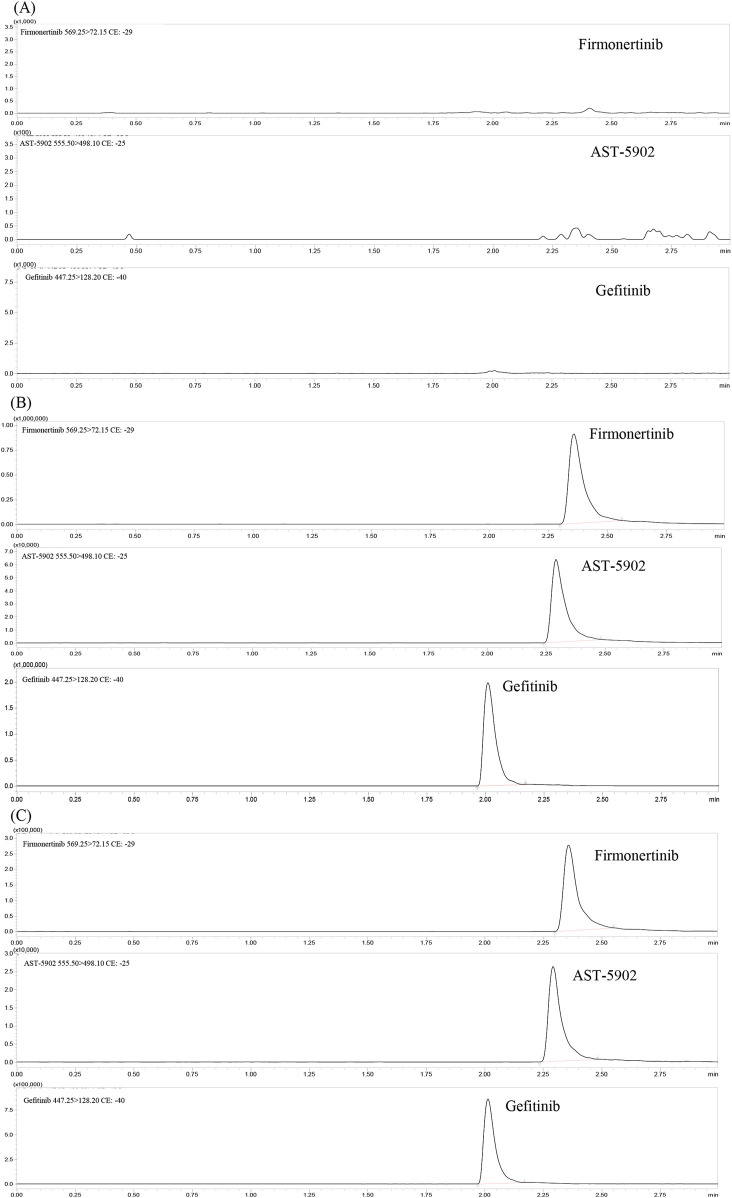
Representative UHPLC–MS/MS chromatograms of firmonertinib, AST-5902 and gefitinib (IS). **(A)** blank plasma; **(B)** a blank plasma sample spiked with firmonertinib, AST-5902 and IS; **(C)** rat plasma sample collected 1 h post oral administration of firmonertinib.

The chromatographic conditions of the whole separation process were optimized in detail, so that the method had better response, higher specificity, shorter detection time and more symmetrical peak shape, thereby achieving effective separation of firmonertinib, AST-5902, and the internal standard (IS). The reverse phase chromatographic column was selected based on the polarities of firmonertinib and AST-5902. The reverse phase chromatographic columns with different column lengths, particle sizes and fillers were compared. The Shim-pack Volex PFPP column (50 mm × 2.1 mm, 1.8 μm) showed good peak profile, separation and retention time. A comprehensive analysis was carried out on various mobile phase combinations composed of acetonitrile, methanol, acetic acid, phosphoric acid and formic acid. The mobile phase, consisting of a methanol solution with 0.1% formic acid and a 0.1% formic acid aqueous solution, demonstrated high separation efficiency and improved peak shape for firmonertinib, AST-5902, and the internal standard. Isocratic and gradient elution, different flow rates from 0.3 to 0.5 mL/min and different column temperatures from 20°C to 40°C were compared. Through a comprehensive comparison of the experimental results, we determined that the optimal conditions were gradient elution mode, a flow rate of 0.4 mL/min, and a column temperature of 35°C. The gradient elution procedure is as follows: Before the start of the elution process, the ratio of 0.1% formic acid-methanol to 0.1% formic acid aqueous solution is 10:90. From 0.5 to 1 min, the volume percentage of 0.1% formic acid-methanol increases to 80%. 1–2 min, the volume percentage of 0.1% formic acid-methanol remains at 80%. From 2 min to 2.5 min, the volumetric percentage of 0.1% formic acid-methanol decreased to 10% and remained at this ratio until the end of the program. The overall runtime of the method is 3 min, with retention times for firmonertinib, AST-5902, and the internal standard (IS) being 2.35 min, 2.28 min, and 2.09 min, respectively. Figure 2 presents the typical chromatograms of blank rat plasma controls, rat plasma spiked with firmonertinib, AST-5902, and IS standards, and rat plasma samples after oral administration of a single dose of firmonertinib.

#### 3.1.2 Mass spectrometer condition optimization

The mass spectrometry parameters such as collision energy, ion detection mode, nebulization gas, and dry gas flow rate were optimized to match the detection of firmonertinib and AST-5902. After optimization, the following mass spectrometry parameters were ascertained: The atomizing gas and drying gas were nitrogen, with their flow rates being 3 L/min and 15 L/min respectively. The interface voltage was 4.5 kV, and the temperature of the heating module was 400°C. Firmonertinib and AST-5902 were detected using the positive ion detection mode, and the fragment ions and collision energy values are presented in Table 1.

#### 3.1.3 Optimization of sample preparation and internal standard

Common approaches for separating target compounds in biological samples are solid-phase extraction, liquid-liquid extraction and protein precipitation. The results of the comparative experiments show that when the plasma samples are treated with the protein precipitation method, the recovery rates of firmonertinib and AST-5902 are both above 90%, and the matrix effect is less than 15%. Therefore, the protein precipitation method is not inferior to the solid-phase extraction method and the liquid-liquid extraction method. Nevertheless, the protein precipitation method excels the other two methods in terms of processing time and simplicity of the method. Consequently, protein precipitation was selected as the pretreatment approach for plasma samples. We compared the protein precipitation effects of organic solvents such as methanol, acetonitrile, and ethanol, salt solutions such as ammonium sulfate and ammonium chloride, and acids such as 10% trichloroacetic acid and 5% sulfosalicylic acid. The results indicated that acetonitrile exhibited a superior protein precipitation effect and more stable chemical properties. Our research results are similar to those from previous studies on the stability of osimertinib. Yuan et al. found in a study on the instability mechanism and pharmacokinetics of osimertinib in plasma that after acetonitrile was added to the plasma containing osimertinib, there was no substantial change in the content of osimertinib in the plasma (Yuan et al., 2022).

To select an appropriate internal standard, we compared classic drugs (sodium phenytoin, diazepam, carbamazepine) with similar drugs (gefitinib, osimertinib, almonertinib). The results showed that gefitinib was suitable for positive ion detection mode, with a retention time similar to that of firmonertinib and AST-5902, it had stable chemical properties and a sensitive response.

### 3.2 Method validation

#### 3.2.1 Selectivity and specificity

Figure 2 presents the typical chromatograms of blank plasma, blank plasma spiked with firmonertinib standard, AST-5902 standard and IS, as well as plasma samples of rats 1 h after oral administration of a single dose of 7.2 mg/kg firmonertinib. The detection results indicate that this method is not interfered by endogenous substances or common reagents.

#### 3.2.2 Linearity and LLOQ

Linear regression was carried out on the area ratios of firmonertinib/AST-5902 to the internal standard versus the corresponding concentrations using the weighted (1/x^2^) least-square. The regression results demonstrated that firmonertinib exhibited an excellent linear relationship within the range of 0.1–500 ng/mL, with the coefficient of determination R^2^ being 0.998. For AST-5902, within the range of 0.05–250 ng/mL, the coefficient of determination R^2^ was 0.996. The LLOQ of firmonertinib was 0.1 ng/mL. The corresponding intra-day CV% and RE% were 5.21% and 1.52%, respectively, and the inter-day CV% and RE% were 2.56% and −0.87%, respectively. The recovery rate was 93.79%. The LLOQ of AST-5902 was 0.05 ng/mL. The corresponding intra-day CV% and RE% were 5.79% and 2.17%, respectively, and the inter-day CV% and RE% were 2.25% and −0.36%, respectively. The recovery rate was 94.95%. Both the precision and accuracy were less than 20%. The precision and accuracy of firmonertinib and AST-5902 were both less than 20%. The signal-to-noise ratios of LLOQ for firmonertinib and AST-5902 were both higher than 5:1. In contrast to previous studies, the LLOQ of firmonertinib and AST-5902 identified in our research were both lower than those utilized for the determination of firmonertinib and AST-5902 in human plasma (Liu et al., 2019).

#### 3.2.3 Recovery and matrix effect

The recovery and matrix effects of firmonertinib and AST-5902 at four different concentrations (LLOQ, low, medium and high) are shown in Table 2, 3. The average recovery rates of firmonertinib at concentrations of 0.1, 0.3, 40, and 400 ng/mL were 93.79%, 95.08%, 96.95%, and 97.84% respectively, and the matrix effects were 93.06%, 97.17%, 93.68%, and 96.94% respectively. The average recovery rates of AST-5902 at concentrations of 0.05, 0.15, 20, and 200 ng/mL were 94.95%, 99.13%, 93.91%, and 96.21% respectively, and the matrix effects were 97.13%, 97.23%, 97.75%, and 98.79% respectively. The recovery rate and matrix effect of IS at the concentration of 300 ng/mL were 90.06% and 97.51% respectively. The results indicated that the average recovery rates of firmonertinib and AST-5902 at LLOQ, low concentration, medium concentration and high concentration were all above 85%, and the matrix effects were all below 15%, suggesting that the recovery rate of this method was relatively high and the matrix effect could be largely ignored.

**TABLE 2:** Recovery and matrix effect of firmonertinib in rat plasma (n = 6).

Analyte	Concentration (ng/mL)	Recovery (%)		Matrix effect (%)	
		Mean ± SD	CV (%)	Mean ± SD	CV (%)
Firmonertinib	0.1	93.79 ± 10.10	10.77	93.06 ± 7.70	8.27
	0.3	95.08 ± 2.75	2.89	97.17 ± 13.16	13.55
	40	96.95 ± 5.67	5.85	93.68 ± 10.40	11.10
	400	97.84 ± 8.07	8.25	96.94 ± 7.71	7.96

**TABLE 3:** Recovery and matrix effect of AST-5902 in rat plasma (n = 6).

Analyte	Concentration (ng/mL)	Recovery (%)		Matrix effect (%)	
		Mean ± SD	CV (%)	Mean ± SD	CV (%)
AST-5902	0.05	94.95 ± 9.28	9.77	97.13 ± 8.95	9.22
	0.15	99.13 ± 10.95	11.04	97.23 ± 8.61	8.85
	20	93.91 ± 6.33	6.75	97.75 ± 11.25	11.51
	200	96.21 ± 9.57	9.94	98.79 ± 5.77	5.84

#### 3.2.4 Accuracy and precision

The method was used to test the quality control samples of four concentrations (LLOQ, low, medium and high) on three different days. The CV%, RE% and recovery rate of the test results were calculated to evaluate the precision and accuracy of the method. The results are presented in Table 4, 5. The intra-day and inter-day CV% of firmonertinib were 5.21%–12.19% and 1.68%–3.77%, respectively. The corresponding intra-day and inter-day RE% were −2.58%–1.52% and −0.87%–0.78%, respectively. The intra-day and inter-day CV% of AST-5902 were 5.79%–8.46% and 2.25%–4.15%, respectively. The corresponding intra-day and inter-day RE% were −2.79%–3.13% and −1.29%–4.31%, respectively. The recovery rates of firmonertinib and AST-5902 were 93.78%–97.84% and 93.91%–99.13% respectively. The CV% and RE% of firmonertinib and AST-5902 at four concentrations were all less than 15%, and the recovery rates were all above 85%. The results demonstrated that this method exhibited excellent precision and accuracy at LLOQ, low concentration, medium concentration and high concentration.

**TABLE 4:** Precision and accuracy for firmonertinib of QC samples in rat plasma (n = 6).

Analyte	Concentration (ng/mL)	Intra-day			Inter-day		
		Mean ± SD	CV (%)	RE (%)	Mean ± SD	CV (%)	RE (%)
Firmonertinib	0.1	0.10 ± 0.01	5.21	1.52	0.10 ± 0.00	2.56	−0.87
	0.3	0.29 ± 0.04	12.19	−2.58	0.30 ± 0.01	3.77	−0.51
	40	39.57 ± 3.09	7.81	−1.08	40.31 ± 0.69	1.68	0.78
	400	393.35 ± 33.97	8.63	−1.66	402.74 ± 9.45	2.35	0.69

**TABLE 5:** Precision and accuracy for AST-5902 of QC samples in rat plasma (n = 6).

Analyte	Concentration (ng/mL)	Intra-day			Inter-day		
		Mean ± SD	CV (%)	RE (%)	Mean ± SD	CV (%)	RE (%)
AST-5902	0.05	0.05 ± 0.00	5.79	2.17	0.05 ± 0.00	2.25	−0.36
	0.15	0.15 ± 0.01	8.31	3.13	0.15 ± 0.01	4.15	−1.29
	20	19.44 ± 1.18	6.05	−2.79	19.78 ± 0.55	2.84	−1.09
	200	201.13 ± 17.02	8.46	0.56	208.63 ± 8.86	4.06	4.31

#### 3.2.5 Stability

The stability of firmonertinib and AST-5902 at three different concentrations (high, medium and low) was determined under four storage conditions. The CV% and RE% of the determination results are calculated to assess the stability of the analyte. The stability determination results of firmonertinib and AST-5902 are presented in Tables 6, 7. The CV% for both firmonertinib and AST-5902 remained below 15%, while the RE% was within ±15% across all tested storage conditions. The experimental results show that firmonertinib and AST-5902 in rat plasma remain stable under both short-term and long-term storage conditions.

**TABLE 6:** Summary of the stability of firmonertinib in rat plasma under different storage conditions (n = 6).

Analyte	Concentration (ng/mL)	Room temperature		4°C		Three freeze-thaw		−80°C	
		RE (%)	RSD (%)	RE (%)	RSD (%)	RE (%)	RSD (%)	RE (%)	RSD (%)
Firmonertinib	0.3	7.47	7.50	−4.98	12.04	−2.83	11.93	3.89	8.70
	40	−0.57	8.22	2.22	4.96	1.26	10.38	4.70	5.41
	400	2.63	12.35	−2.90	8.42	−2.69	7.29	1.83	9.41

**TABLE 7:** Summary of the stability of AST-5902 in rat plasma under different storage conditions (n = 6).

Analyte	Concentration (ng/mL)	Room temperature		4°C		Three freeze-thaw		−80°C	
		RE (%)	RSD (%)	RE (%)	RSD (%)	RE (%)	RSD (%)	RE (%)	RSD (%)
AST-5902	0.2	4.37	5.04	−1.94	7.72	1.93	4.99	0.97	7.49
	5	1.25	9.12	−1.36	4.04	5.50	7.93	3.06	6.30
	40	0.31	8.03	0.00	3.33	−0.91	10.10	−0.53	6.89

### 3.3 Application of the method in DDI study between firmonertinib and paxlovid

**FIGURE 3:**
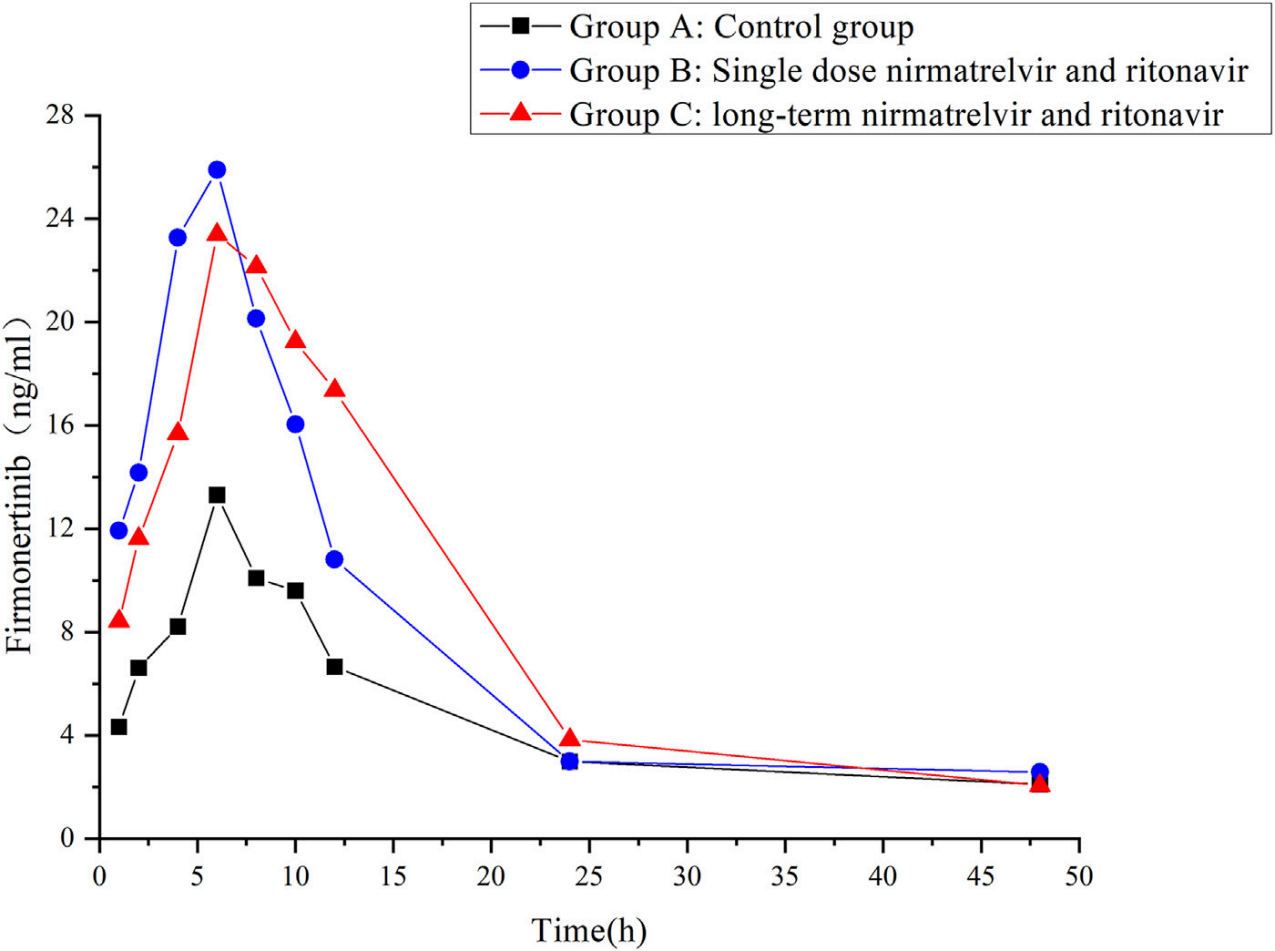
Mean plasma concentration-time profiles of firmonertinib in different treatment groups of rats. Group A: control group; Group B: single dose of 55 mg/kg nirmatrelvir and 20 mg/kg ritonavir; Group C: long-term administration of 55 mg/kg nirmatrelvir and 20 mg/kg ritonavir (n = 6, mean).

**FIGURE 4:**
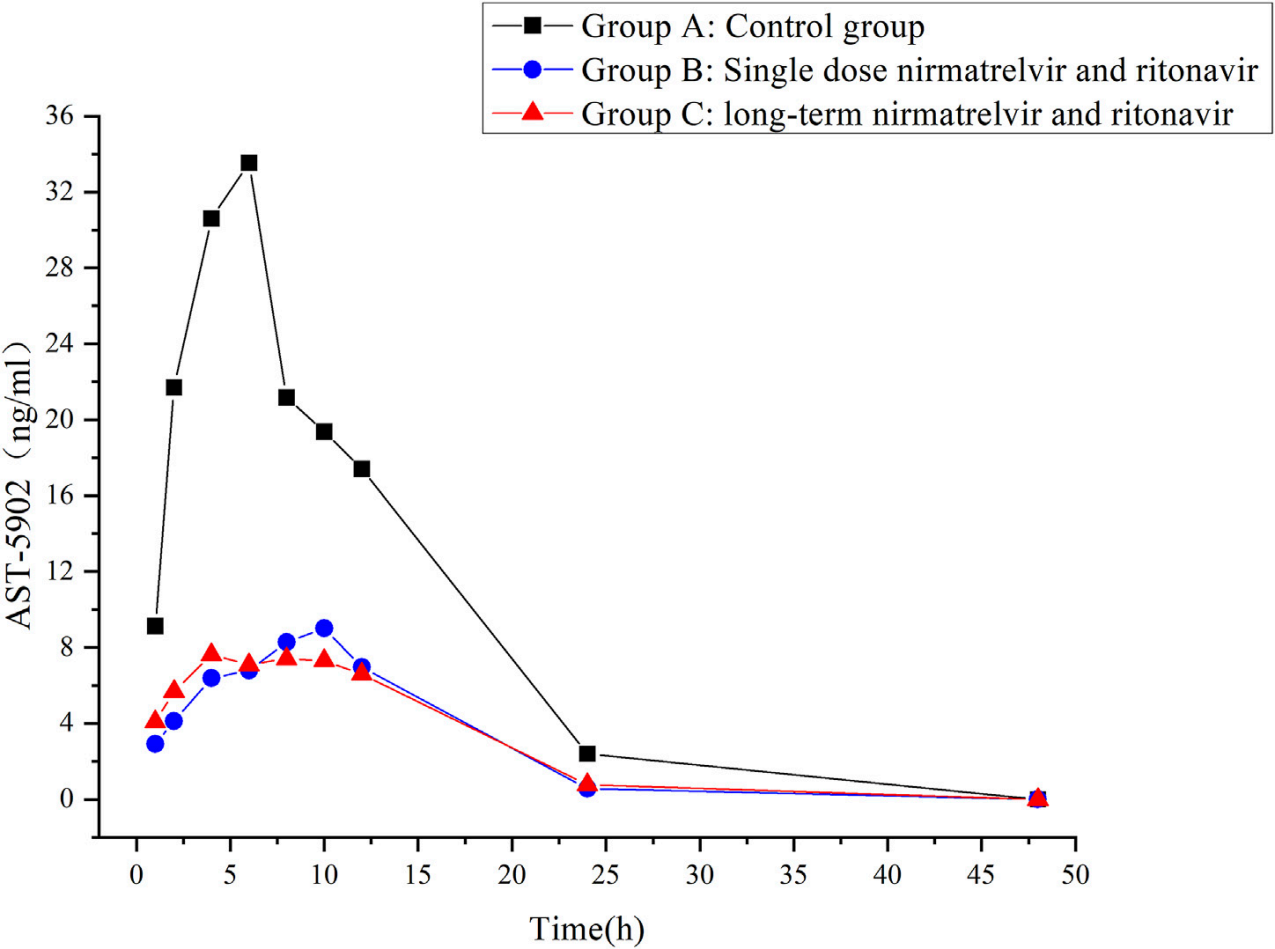
Mean plasma concentration-time profiles of AST-5902 in different treatment groups of rats. Group A: control group; Group B: single dose of 55 mg/kg nirmatrelvir and 20 mg/kg ritonavir; Group C: long-term administration of 55 mg/kg nirmatrelvir and 20 mg/kg ritonavir (n = 6, mean).

The validated UHPLC–MS/MS method was employed to study the interaction between firmonertinib and paxlovid in SD rats. The average plasma concentration-time curves of firmonertinib and its metabolite AST-5902 in each group of rats after a single intragastric administration of 7.2 mg/kg firmonertinib are presented in Figures 3, 4. The pharmacokinetic data of firmonertinib and its metabolite AST-5902 derived from the non-compartmental model analysis of plasma concentration-time curves using DAS software, are summarized in Table 8, 9.

**TABLE 8:** The main pharmacokinetic parameters of firmonertinib in different treatment groups of rats. Group A: control group (single-dose 7.2 mg/kg firmonertinib), Group B: single dose administration group (single-dose 55 mg/kg nirmatrelvir and 20 mg/kg ritonavir) and Group C: long-term administration group (5 days, twice a day of 55 mg/kg nirmatrelvir and 20 mg/kg ritonavir). (n = 6, mean ± SD).

Parameters	Unit	Group A	Group B	Group C
AUC_(0-t)_	µg/L*h	270.14 ± 28.44	456.29 ± 124.74*	421.10 ± 21.43*
AUC_(0-∞)_	µg/L*h	367.39 ± 89.65	485.84 ± 102.79*	458.07 ± 46.00*
MRT_(0-t)_	h	24.46 ± 2.43	19.22 ± 2.34*	19.59 ± 2.48*
MRT_(0-∞)_	h	61.77 ± 44.69	28.36 ± 6.88*	30.28 ± 5.96*
t_1/2_	h	39.71 ± 35.66	16.22 ± 6.77*	17.88 ± 5.91*
T_max_	h	6.33 ± 1.97	6.00 ± 2.19	5.00 ± 1.10
V_z/F_	L/kg	1,012.75 ± 591.14	374.40 ± 209.49*	399.86 ± 95.81*
CL_z/F_	L/h/kg	20.45 ± 4.27	15.33 ± 2.91*	15.85 ± 1.56*
C_max_	ng/mL	13.54 ± 4.43	26.96 ± 9.75*	27.88 ± 4.22*

Notes: Compared with Group A, *P < 0.05.

Abbreviations: AUC, area under the curve; MRT, mean retention time.

**TABLE 9:** The main pharmacokinetic parameters of AST-5902 in different treatment groups of rats. Group A: control group (single-dose 7.2 mg/kg firmonertinib), Group B: single dose administration group (single-dose 55 mg/kg nirmatrelvir and 20 mg/kg ritonavir) and Group C: long-term administration group (5 days, twice a day of 55 mg/kg nirmatrelvir and 20 mg/kg ritonavir). (n = 6, mean ± SD).

Parameters	Unit	Group A	Group B	Group C
AUC_(0-t)_	µg/L*h	387.45 ± 58.17	122.32 ± 14.99*	122.20 ± 12.76*
AUC_(0-∞)_	µg/L*h	404.74 ± 60.36	184.95 ± 147.48*	125.17 ± 13.15*
MRT_(0-t)_	h	8.37 ± 0.39	8.94 ± 0.62	9.22 ± 0.19
MRT_(0-∞)_	h	9.35 ± 0.40	8.73 ± 2.41	9.70 ± 0.33
t_1/2_	h	4.94 ± 0.98	6.14 ± 4.31	3.65 ± 0.58
T_max_	h	5.33 ± 1.03	8.33 ± 2.94*	8.67 ± 1.63*
V_z/F_	L/kg	129.48 ± 35.51	361.75 ± 62.92*	306.26 ± 56.68*
CL_z/F_	L/h/kg	18.13 ± 2.72	51.06 ± 18.84*	58.13 ± 7.01*
C_max_	ng/mL	39.04 ± 2.46	9.79 ± 1.70*	10.18 ± 1.42*

Notes: Compared with Group A, *P < 0.05.

Abbreviations: AUC, area under the curve; MRT, mean retention time.

The experimental results showed that compared with the firmonertinib monotherapy group, when firmonertinib was combined with nirmatrelvir and ritonavir, whether it was a single dose of nirmatrelvir and ritonavir or a long-term dose of nirmatrelvir and ritonavir (5 days), the main pharmacokinetic parameters of firmonertinib, such as AUC_(0-t)_, AUC_(0-∞)_ and C_max_, were significantly increased (P < 0.05), while MRT_(0-t)_, MRT (_0-∞)_, t_1/2_, V_z/F_, and CL_z/F_ were significantly decreased (P < 0.05). Specifically, AUC_(0-t)_ increased by 1.68 and 1.55 times, AUC_(0-∞)_ increased by 1.32 and 1.24 times, and C_max_ increased by 1.99 and 2.05 times, respectively. Meanwhile, MRT_(0-t)_ decreased by 22% and 20%, MRT_(0-∞)_ decreased by 55% and 51%, t_1/2_ decreased by 60% and 55%, V_z/F_ decreased by 64% and 61%, and CL_z/F_ decreased by 26% and 23%, respectively. Pharmacokinetic parameters of AST-5902, the metabolite of firmonertinib, showed that compared with the firmonertinib monotherapy group, when firmonertinib was combined with nirmatrelvir and ritonavir, whether in the single-dose nirmatrelvir and ritonavir group or the long-term nirmatrelvir and ritonavir group, the main pharmacokinetic parameters of AST-5902, such as AUC_(0-t)_, AUC_(0-∞)_ and C_max_, were significantly decreased (P < 0.05), while T_max_, V_z/F_, and CL_z/F_ were significantly increased (P < 0.05). Specifically, AUC_(0-t)_ decreased by 69%, AUC_(0-∞)_ decreased by 55% and 70%, and C_max_ decreased by 75% and 74%, respectively. Meanwhile, T_max_ increased by 1.56 times and 1.62 times, V_z/F_ increased by 2.79 times and 2.36 times, and CL_z/F_ increased by 2.81 times and 3.2 times, respectively.There were no significant differences in the main pharmacokinetic data of firmonertinib and AST-5902 between the single-dose nirmatrelvir and ritonavir group and the long-term nirmatrelvir and ritonavir group (P > 0.05).

Drug-drug interaction (DDI) refers to the phenomenon in which the effects of drugs are altered when two or more drugs are administered concurrently or sequentially (McQuade and Campbell, 2021). When DDIs occur, the conventional dosage of drugs may increase the exposure of drugs in the body or produce a synergistic effect, or it may reduce the exposure of drugs in the body or produce an antagonistic effect, thereby resulting in the occurrence of adverse drug reactions or treatment failure (Garrison et al., 2018; Yu et al., 2023; Lee et al., 2024). A study on cancer patients receiving anti-cancer drug treatment shows that the number of potential DDIs increases significantly with the rise in the number of comorbidities and concomitant medications (Koni et al., 2022). Therefore, the assessment of DDIs is an important aspect in evaluating the benefits and risks of combined medication use in clinical practice (Sachdev and Gupta, 2019). As a novel third-generation epidermal growth factor receptor TKI targeting EGFR sensitive mutations, firmonertinib has received relatively little research on DDIs. A previous clinical study evaluating the impact of itraconazole (CYP3A4 inhibitor) on the pharmacokinetics of firmonertinib have demonstrated that co-administration with itraconazole significantly increases the exposure of firmonertinib. A higher exposure of firmonertinib would significantly reduce the concentration of its active metabolite AST-5902, thereby potentially increasing the risk of serious adverse reactions such as QT interval prolongation and interstitial pneumonia (Heng et al., 2021). Another clinical study evaluating the effect of rifampicin on the pharmacokinetics of firmonertinib demonstrated that when firmonertinib was co-administered with therapeutic doses of rifampicin (strong CYP3A4 inducer), the exposure of firmonertinib in the body was significantly reduced, which might lead to treatment failure (Zhu et al., 2021). These examples remind us that when firmonertinib is used in combination with other drugs, especially with inhibitors or inducers of CYP3A4, the occurrence of DDIs must be noted. Otherwise, adverse consequences such as serious adverse drug reactions or treatment failure may arise.

Over the past several years, the entire world has been impacted by COVID-19. Paxlovid ranks among the most frequently utilized antiviral medications in the treatment of COVID-19 (Najjar-Debbiny et al., 2023). Paxlovid consists of nirmatrelvir and ritonavir. In this combination, only nirmatrelvir has antiviral activity. Ritonavir, as a strong inhibitor of CYP3A4, when used in combination with nirmatrelvir, can increase the exposure of nirmatrelvir in the body and enhance its efficacy against SARS-CoV-2 (Marzi et al., 2022). Owing to the application of ritonavir, there is a high probability that nirmatrelvir/ritonavir will give rise to clinically significant DDIs when combined with other medications. Previous studies have reported that the concentration of tacrolimus in patients undergoing Paxlovid treatment after organ transplantation would increase significantly, resulting in the occurrence of acute kidney injury and mental symptoms (Prikis and Cameron, 2022; Liu et al., 2023; Michael et al., 2023). Nieminen et al. research indicates that when oxycodone is administered simultaneously with ritonavir, the AUC of oxycodone triples. The increase in drug exposure might result in the occurrence of adverse reactions like nausea, vomiting, and respiratory depression (Nieminen et al., 2010). The research findings of Boosman et al. indicate that the pharmacokinetic exposure of erlotinib in the body when 75 mg of erlotinib is administered orally in combination with ritonavir is comparable to that when 150 mg of erlotinib is taken orally (Boosman et al., 2022). Despite the abundance of information regarding drug interactions of Paxlovid, the majority is deduced from previous interaction studies with ritonavir. Direct research on the interactions between Paxlovid and other drugs remains relatively scarce. Therefore, the current knowledge and experience in treating SARS-CoV-2 infected patients with underlying conditions using Paxlovid are still limited.

Cancer patients represent one of the populations most susceptible to infection with COVID-19 (Liang et al., 2020). Statistics indicate that the incidence of COVID-19 among cancer patients is approximately 1%–6%, far exceeding that among the general population and patients with other underlying diseases (Miyashita et al., 2020; Zhang et al., 2020). Reports from New York (Miyashita et al., 2020), Italy (Fratino et al., 2020) and China (Dai et al., 2020; Zhang et al., 2020) all indicate that cancer patients have a worse prognosis after being infected with COVID-19. Among cancer patients with COVID-19, 39% may experience severe adverse outcomes, such as being admitted to the ICU, requiring mechanical ventilation or death, with a mortality rate as high as 30% (Zhang et al., 2020). Among all cancer patients, lung cancer patients are exposed to a significantly higher risk of severe COVID-19 outcomes. The possible causes for this might be the impaired immunity resulting from the anti-tumor treatments received by lung cancer patients, as well as the lung damage and reduced lung capacity caused by the cancer itself (Garassino et al., 2020; Monari et al., 2021). Furthermore, patients with lung cancer typically have concomitant pulmonary and cardiovascular diseases, and the majority of them are smokers. In conclusion, lung cancer patients have almost all the factors related to a worse prognosis in COVID-19 (Guan et al., 2020; Richardson et al., 2020; Wu et al., 2020; Zhou et al., 2020).

During the COVID-19 pandemic, not only do cancer patients have the risk of being infected with COVID-19 and suffering from severe adverse prognoses, but the delays in the diagnosis and treatment of cancer can also exert an extremely significant negative influence on the cancer progression of patients. The strict epidemic control measures taken by the government and hospital management departments to reduce the risk of COVID-19 infection often delay the diagnosis and treatment of lung cancer patients (Melidis and Vantsos, 2020). During the early stage of the COVID-19 outbreak in China, cancer patients were required to complete the following processes before being admitted to hospital for treatment: temperature measurement, SARS-CoV-2 viral nucleic acid detection of throat swab specimens, and chest CT (Ge, 2023; Li R. et al., 2023). These epidemic prevention measures resulted in many patients altering or even being forced to suspend their originally scheduled treatment plans during the epidemic prevention and control period. A study conducted by London et al. using the COVID-19 and Cancer Research Network (CCRN) revealed that during the COVID-19 pandemic, the attendance rate of all cancer-related patients dropped significantly compared to the pre-pandemic period (London et al., 2020). Rodriguez et al.’s study on cancer patients in the United States shows that almost more than half of the patients changed their treatment plans due to the impact of the epidemic (Rodriguez et al., 2021). Regardless of the stage of cancer, the delay in cancer treatment for patients exceeded 4 weeks (Rodriguez et al., 2021). The delays in diagnosis and disruptions in treatment caused by the epidemic will both bring negative clinical consequences to cancer patients.

It is of critical importance to concurrently administer antiviral and anti-tumor therapies in NSCLC patients infected with COVID-19. Therefore, the probability of patients using both firmonertinib and Paxlovid simultaneously has increased significantly. However, there are no reports on the drug interaction between firmonertinib and Paxlovid at present. Previous studies have shown that the contribution of CYP3A4 to drug metabolism is crucial for predicting the degree of DDIs (Zhou, 2008). Drugs mainly metabolized by CYP3A4 (sensitive substrates of CYP3A4) are expected to be severely affected by ritonavir. Considering that the principal metabolic enzyme of firmonertinib is CYP3A4, some potential DDIs might occur between firmonertinib and Paxlovid. The research data of this study truly presented the DDIs between firmonertinib and Paxlovid in rats. Compared with the group using firmonertinib alone, the pharmacokinetic parameters related to drug exposure of firmonertinib in the group co-administered with Paxlovid in rats were significantly increased. The peak concentration and AUC of the active metabolite AST-5902 of firmonertinib in rat plasma in the combination drug group were significantly lower than those in the control group, indicating that the metabolites generated in the rats was significantly reduced after co-administration with Paxlovid. The pharmacokinetic data of firmonertinib and AST-5902 can essentially corroborate each other. These data reveal that Paxlovid can significantly increase the systemic exposure of firmonertinib in rats. As the main metabolic pathway of firmonertinib is through CYP3A4 metabolism, we speculate that the possible mechanism is that Paxlovid inhibit the activity of CYP3A4 enzyme, thereby suppressing the metabolism of firmonertinib. However, when comparing a single dose of Paxlovid with a 5-day consecutive administration of Paxlovid, there were no significant differences in the pharmacokinetic data of firmonertinib and its metabolites. This indicates that the degree of metabolic inhibition of firmeritinib by Paxlovid is not related to the duration of Paxlovid administration. Therefore, in clinical practice, when firmonertinib is combined with Paxlovid, according to our research findings, the dose of firmonertinib might need to be decreased. Doctors and pharmacists should closely monitor the potential adverse reactions of firmonertinib. If necessary, therapeutic drug monitoring (TDM) of firmonertinib should be carried out, and the plasma concentration should be taken as the basis for dose adjustment.

Although our results confirmed the DDIs between firmonertinib and Paxlovid, this study still has several limitations. Firstly, in all experiments, nirmatrelvir and ritonavir were regarded as inhibitors, while firmonertinib was considered as the substrate. Previous studies have shown that firmonertinib is both a substrate and an inducer of CYP3A4 (Liu et al., 2020). Therefore, we are not sure whether firmonertinib would exert an inducing effect on the metabolism of nirmatrelvir and ritonavir if nirmatrelvir and ritonavir were taken as substrates. Secondly, we used healthy rats as the research subjects and did not utilize a lung cancer animal model to study the pharmacokinetic interaction between firmonertinib and Paxlovid. The pharmacokinetic results of the same drug in healthy rats and lung cancer animal models may be different. Finally, due to the species differences between rats and humans, all the research results can only provide references for clinical practice. Further clinical studies are needed to ultimately confirm the DDIs between firmonertinib and Paxlovid in patients with advanced NSCLC.

## 4 Conclusion

In this study, an accurate, stable, rapid and simple UHPLC-MS/MS method for the simultaneous determination of firmonertinib and its metabolite AST-5902 in rat plasma was established and validated. Through the optimization of method parameters, lower LLOQ (0.1 ng/mL for firmonertinib and 0.05 ng/mL for AST-5902), extensive standard curve (0.1–500 ng/mL for firmonertinib and 0.05–250 ng/mL for AST-5902), shorter detection time (3 min), and a facile sample processing method were achieved. The established detection method was successfully applied to the study of drug interactions between firmonertinib and Paxlovid in rats. The results demonstrate that Paxlovid have significant inhibitory effect on the metabolism of firmonertinib. When used in combination, it will increase the *in vivo* exposure of firmonertinib, which may lead to serious adverse reactions. Considering the complexity of patients with advanced malignant tumors, corresponding clinical studies should be further carried out to validate the results of animal experiments.

## Data Availability

The original contributions presented in the study are included in the article/supplementary material, further inquiries can be directed to the corresponding author.
